# Gemcitabine, dexamethasone, and cisplatin (GDP) chemotherapy with sandwiched radiotherapy in the treatment of newly diagnosed stage IE/IIE extranodal natural killer/T‐cell lymphoma, nasal type

**DOI:** 10.1002/cam4.2214

**Published:** 2019-05-02

**Authors:** Shu Tian, Ruichen Li, Tian Wang, Shengzi Wang, Rong Tao, Xichun Hu, Hao Ding

**Affiliations:** ^1^ Department of Radiation Oncology, Eye Ear Nose and Throat Hospital Fudan University Shanghai China; ^2^ Department of Medical Oncology Fudan University Shanghai Cancer Center Shanghai China; ^3^ Department of Oncology Shanghai Medical College, Fudan University Shanghai China; ^4^ Department of Hematology Xinhua Hospital, Shanghai Jiaotong University School of Medicine Shanghai China

**Keywords:** chemotherapy, extranodal natural killer/T cell lymphoma, nasal‐type, GDP regimen, gemcitabine, radiotherapy

## Abstract

Extranodal natural killer/T‐cell lymphoma (ENKL), nasal‐type is a rare but highly aggressive disease with poor prognosis. Optimal treatment strategies for newly diagnosed localized ENKL have not been fully defined. Here we retrospectively analyzed 72 patients with newly diagnosed stage IE/IIE ENKL treated with gemcitabine, dexamethasone, and cisplatin (GDP) regimen chemotherapy with sandwiched radiotherapy in our department between May 2012 and September 2014. After 2 cycles of GDP induction chemotherapy, the complete response rate (CRR) and overall response rate (ORR) were 30.6% (22/72) and 91.7% (66/72). After whole treatment completion, the CRR and ORR were 81.9% (59/72) and 91.7% (66/72), respectively. With a median follow‐up of 57.8 months (Interquartile Range 54.0‐64.5 months), the 5‐year progression‐free survival rate was 70.9% (95% CI, 60.1% to 81.7%), and the 5‐year overall survival rate was 72.0% (95% CI, 61.6% to 82.4%), respectively. Patients with CRR after treatment had better prognosis than their counterparts. The major adverse events were myelosuppression, liver dysfunction, gemcitabine‐related skin rash, and digestive tract toxicities. Grade 3 to 4 neutropenia and thrombocytopenia were 18.0% (13/72) and 15.3% (11/72), respectively. No treatment related deaths were observed. It is concluded that the GDP regimen with sandwiched radiotherapy was an effective and well‐tolerated treatment for newly diagnosed stage IE/IIE ENKL, nasal‐type.

## 1 INTRODUCTION

Extranodal natural‐killer (NK)/T‐cell lymphoma (ENKL), nasal‐type is a rare highly aggressive and heterogeneous disease with a geographical and racial predilection for Asian and South American populations.^1^ In a retrospective survey with a single institution experience in China, ENKL accounted for 17.1% of all non‐Hodgkin lymphomas.^2^ ENKL most often originates in the nasal and upper aerodigestive tract, but can also arise in the skin, soft tissue, gastrointestinal tract, and testis. Epstein‐Barr virus (EBV) may play a role in the development of this entity.^3^ Despite two thirds of ENKL patients are diagnosed with early‐stage (Ann Arbor Stage I or II), it shows a highly aggressive clinical course and poor prognosis, with 5‐year overall survival ranging from 34% to 89%.^1^, ^3^

Radiotherapy (RT) has been recognized as the backbone of curative intent for early‐stage ENKL, with a total dose of more than 50 Gy.^4^, ^5^ However, the systemic relapse was as high as 25% to 40%,^6^ suggesting that addition of chemotherapy (CT) might contribute to reduce systemic dissemination in localized ENKL. ENKL tends to be resistant to conventional anthracycline‐based CT regimen, such as CHOP or CHOP‐like regimens, partly due to a multidrug resistance (MDR) phenotype.^7^ The addition of conventional CT to RT has failed to reduce systemic failure and improve overall survival (OS) or progression‐free survival (PFS) in early‐stage ENKL patients. Although L‐asparaginase based CT regimens are effective in relapsed and refractory ENKL patients in improving complete remission (CR) rate and OS, their toxicities are nearly unacceptable.^8^, ^9^

Gemcitabine is a pyrimidine antimetabolite with a chemical structure similar to cytosine arabinoside, that has more effective cellular kinetics, including intracellular incorporation, phosphorylation, and retention.^10^ Several studies have demonstrated that the gemcitabine alone and/or containing regimen had high clinical activity and low toxicity both in the treatment of T‐cell unspecified lymphoma^11^, ^12^ and in refractory or relapsed NK/T‐cell lymphoma.^13^, ^14^ Meanwhile, the sandwich combined modality therapy has achieved an excellent outcome for localized ENKL with acceptable toxicity.^15^, ^16^, ^17^, ^18^ Here, we reported a retrospective study evaluating the efficacy and safety profiles of the sandwich protocol in 72 patients with newly diagnosed stage IE/IIE ENKL, nasal type: an initial 2 cycles of gemcitabine, dexamethasone, and cisplatin (GDP), followed by earlier involved‐field radiation therapy (IFRT), and further 2 to 4 cycles consolidation CT.

## 2 MATERIALS AND METHODS

### 2.1 Eligibility criteria

From May 2012 to September 2014, patients with newly diagnosed Ann Arbor stage I or II ENKL having fever and/or extensive lesions were enrolled. Diagnosis of ENKL with typical morphology and immunophenotype according to the 2016 WHO classification of lymphomas.^19^ Primary site of disease was required to occur within the upper aerodigestive tract. The pretreatment clinical staging evaluation consisted of a history taking, physical examination, complete blood count, serum biochemistry, lactate dehydrogenase (LDH), beta 2‐microglobulin, bone marrow aspiration, and/or biopsy. Computed tomography of nasal cavity, neck, chest, abdomen, and pelvis; magnetic resonance imaging (MRI) of nasal cavity and a positron emission tomography scan with diagnostic quality computed tomography (PET‐CT) were recommended but not mandatory. The Korean prognostic index (KPI) was analyzed to assess prognosis.^20^ Primary tumor invasion (PTI) was defined as the presence of primary disease that extended into neighboring structures or organs, or the involvement of multiple, contiguous primary sites, regardless of either the stage or primary site. The primary site was defined according to the anatomical site of origin, including the nasal cavity, nasopharynx, oropharynx, hypopharynx, and other extranodal sites. The study was conducted according to the Declaration of Helsinki and the Guidelines for Good Clinical Practice. Written informed consent was obtained from all participants.

### 2.2 Treatment protocol

This study was conducted of “sandwich” protocols. The patients were initially treated with 2 cycles of GDP regimen repeated every 21 days. The GDP doses and administration schedule were as follows: gemcitabine 800‐1000 mg/m^2^ intravenously on day 1 and 8, dexamethasone 40 mg intravenously on days 1‐4, cisplatin 25 mg/m^2^ intravenously on days 1‐3. IFRT was started after 2 cycles of chemotherapy, and further 2 to 4 cycles of consolidation chemotherapy were finished after completion of radiotherapy. The doses of gemcitabine and cisplatin were decreased 20% in the next cycle if grade 3 to 4 hematological toxicity was observed. Patients without tumor response (complete response or partial response) after 2 cycles were treated with radical radiation immediately. HBs‐Ag and HBV‐DNA copy number were monitored routinely and entecavir was administrated to patient with HBs‐Ag positive.

Patients were treated with a three‐dimensional conformal radiotherapy by using 6 MV linear accelerator by conventional fractionation schedule (1.8‐2.0 Gy/fraction, 5 fractions/week). The gross target volume (GTV) contained primary tumors and positive cervical nodes identified in pre‐chemotherapy images and physical examination. The clinical target volume (CTV) contained the tumor‐adjacent tissues prior to the chemotherapy. In patients with nasal lymphoma limited to the anterior part of the nasal cavity, the CTV of extended field RT encompasses the bilateral nasal cavity, frontal ethmoid sinus, and ipsilateral maxillary sinus. In patients with lymphoma located to the posterior part of the nasal cavity, the CTV was extended to include the above‐mentioned and nasopharynx. In patients with lymphoma involved nasopharynx, the Waldeyer's ring was included in the radiation fields. Prophylactic cervical lymph node irradiation was generally not performed for stage I patients. All stage II patients with positive cervical nodes received neck irradiation. The planning target volume (PTV) was confined to 3‐mm area outside the CTV. A total dose of 54‐56 Gy was given to the GTV, a dose not less than 50 Gy was given to PTV.

### 2.3 Response and safety assessment

The treatment responses were assessed at 3 points according to the International Working Group Recommendation for Response Criteria for non‐Hodgkin's lymphoma,^21^, ^22^ including 2 weeks after the second cycle of the GDP regimen, post radiotherapy and 1 month after the treatment completion. The primary endpoints were CR and objective response rate (ORR) after initial chemotherapy and whole treatment. Secondary endpoints were OS, PFS, and toxicity. All adverse reactions were graded according to the National Cancer Institute Common Terminology Criteria for Adverse Events (NCI‐CTCAE) Version 4.0.

### 2.4 Statistical analysis

OS was measured from the date of diagnosis to death from any cause or the date of the last follow‐up. PFS was calculated as diagnosis to first progression, relapse after response, or death from any cause, or the date of last follow‐up. The chi‐square test was used to calculate statistical group comparisons of categorical variables. PFS and OS were estimated by the Kaplan‐Meier method. Univariate analysis of prognostic factors was estimated using the log‐rank test. Multivariate analysis was performed using the Cox proportional hazard model. Two‐sided *P* < 0.05 was considered significant. All analyses were performed using SPSS Statistics 22.0 software (SPSS Inc, Chicago, IL).

## 3 RESULTS

### 3.1 Patient characteristics

The clinical characteristics of 72 patients were shown in Table 1. There was a gender predominance, with a male‐to‐female ratio 2.1:1. The median age was 52.5 years (range, 21‐79), with 16 patients (22.2%) being older than 60 years. Fifty‐four (75%) patients initially presented as stage I and 18 (25%) patients as stage II. Most patients had good performance status: an Eastern Cooperative Oncology Group (ECOG) Performance Status (PS) of 0 or 1, while 62.5% patients had B symptoms at presentation. Primary anatomic sites were nasal cavity (n = 63), nasopharynx (n = 5), oropharynx (n = 3), hypopharynx (n = 1). PTI was observed in 48 (66.7%). A total of 22 (30.6%) patients were included in the intermediate‐high and high‐risk groups according to KPI.

**Table 1:** The clinical characteristics for all patients at baseline

Characteristic		No. of patients	%
Age (y)	Median (range)	52.5 (21‐79)	—
	>60	16	22.2
Sex	Male	49	72.2
	Female	23	36.1
B symptoms	No	27	37.5
	Yes	45	62.5
Elevated LDH	No	50	69.4
	Yes	22	30.6
ECOG PS	0‐1	66	91.7
	2	6	8.3
Primary site	Nasal primary site	63	87.5
	Non‐nasal primary site	9	12.5
PTI	No	24	33.3
	Yes	48	66.7
Ann Arbor stage	I	54	75.0
	II	18	25.0
LN involvement	No	57	79.2
	Yes	15	20.8
KPI	0	22	30.6
	1	28	38.9
	2	16	22.2
	3	6	8.3

Abbreviations: ECOG, Eastern Cooperative Oncology Group; KPI, Korean Prognostic Index; LDH, lactate dehydrogenase; LN, lymph node. PTI, primary tumor invasion; PS, performance status.

### 3.2 Response to treatment

**Figure 1:**
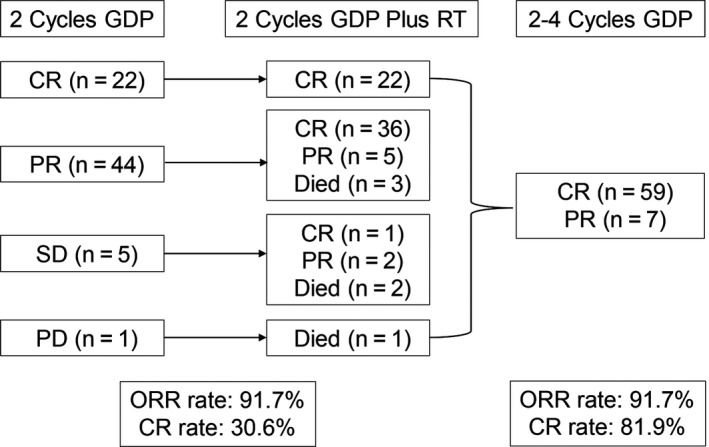
Responses to treatment

The responses to the treatment were presented in Figure 1. The total cycles of GDP regimen received by 72 patients were 142 before RT, with a median of 2 cycles (range, 2 cycles). Two patients were referred to RT after only 1 cycle of CT, 1 for rapid disease progression and the other for delayed recovery of liver function. Then, patients received a median radiation dose of 54 Gy (range, 7.2‐56 Gy). Six patients died during RT due to rapid disease progression or lymphoma‐associated hemophagocytic syndrome (HPS). The remaining 66 (91.7%) patients completed the planned sandwich chemoradiation. After RT, they totally received 140 cycles of GDP regimen with a median of 2 cycles (range, 2‐4 cycles). After 2 cycles of GDP regimen, there were 22 (30.6%) patients in CR, 44 (61.1%) in partial remission (PR), 5 (6.9%) in stable disease (SD), and 1 (1.4%) in progressive disease (PD). Thirty‐six of 45 patients (80%) having B symptoms got fever remission after initial CT. At the completion of RT, 22 patients remained in CR, 26 patients with PR, and 1 with SD converted to CR. The subsequent CT after RT did not change the response rate. That was, after GDP in combination with RT, 59 (81.9%) and 7 (9.7%) patients achieved CR and PR, respectively. Therefore, the ORR was 91.7% both after 2 cycles of initial CT and at the end of treatment.

### 3.3 Relapse and survival

**Figure 2:**
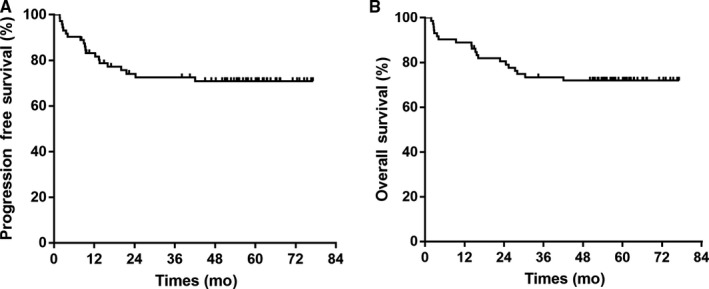
Kaplan‐Meier estimates of survival of 72 patients treated with GDP chemotherapy with sandwiched radiotherapy. (A) Progression‐free survival. (B) Overall survival

With a median follow‐up of 57.8 months (Interquartile Range 54.0‐64.5 months), the 5‐year PFS and OS were 70.9% (95% CI, 60.1% to 81.7%) and 72.0% (95% CI, 61.6% to 82.4%), respectively (Figure 2). The median PFS and OS points have been not reached. During the follow‐up period, 30.1% (22/72) patients developed treatment failure. Among them, 8 patients had local failure and 16 patients developed systemic failure. Local recurrences were located outside the prior radiation field in 6 cases and in the radiation field in 2 cases received more than 50 Gy. In systemic failure patients, the involved sites were skin, bone marrow, ileocecum, breast, pelvic cavity, and inguinal lymph nodes. All the patients received salvage therapy, 11 patients with treatment failure received MEDA salvage regimen (methotrexate, etoposide, dexamethasone, and pegaspargase) in hematological department,^23^ 2 patients subsequently received autologous hematopoietic stem cell transplantation (HSCT), 5 of them achieved CR and were alive with no evidence of disease.

### 3.4 Univariate and multivariate analysis

**Figure 3:**
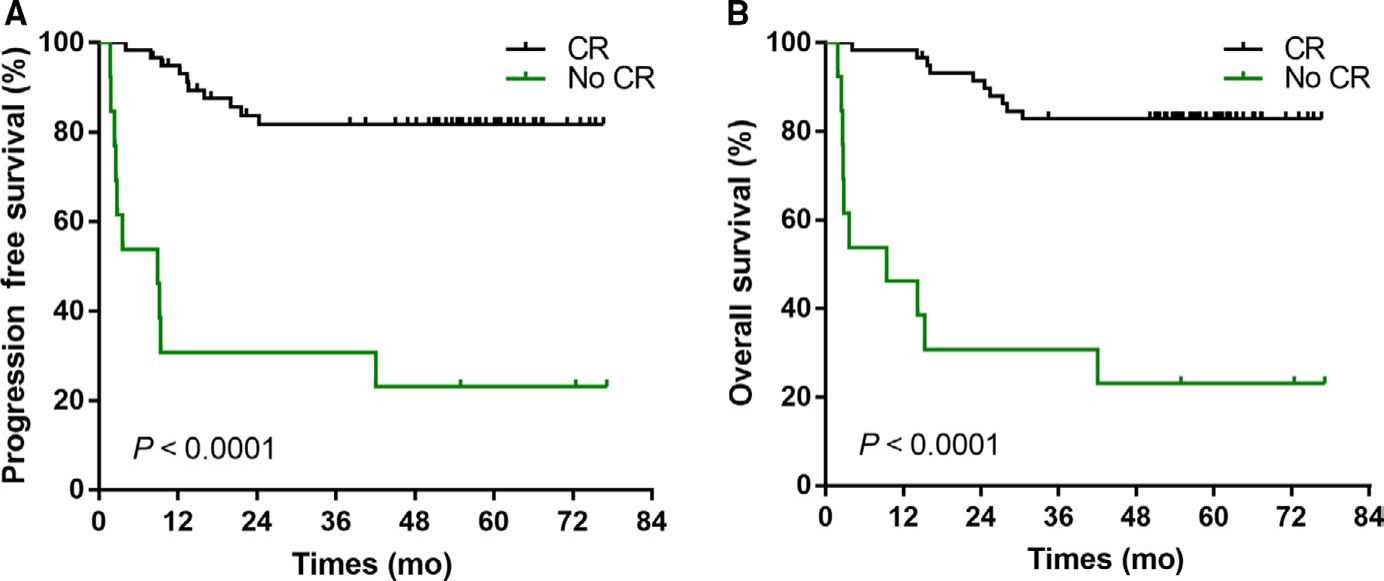
Effect of complete response (CR) at the end of treatment on progression‐free survival (A) and overall survival (B) of patients with early stage ENKL

The clinical factors predicting survival at univariate analysis were as follows: age, sex, B symptoms, LDH level, ECOG PS, PTI, stage, lymph‐node (LN) involvement, KPI, response to initial CT, and treatment completion (Table 2). The factors associated with reduced PFS and OS were the presence of B symptoms and PTI, evaluated LDH level, ECOG PS of 2, KPI of 2 to 3 and no CR to the whole treatment (*P* < 0.001, Figure 3). In a multivariate analysis with Cox regression model, PFS and OS were significantly associated with ECOG PS (hazard ratio, 7.909 and 10.557), and response to completion treatment (hazard ratio, 4.104 and 3.682) (Table 3).

**Table 2:** Clinical factors for survival in univariate analysis

Factor		5‐y PFS		5‐y OS	
		% (95% CI)	*P* value	% (95% CI)	*P* value
Age (y)	≤60	71.6 (59.4‐83.8)	0.661	73.0 (61.2‐84.8)	0.680
	>60	68.2 (45.1‐91.3)		68.8 (46.1‐91.5)	
Sex	Male	76.6 (64.4‐88.8)	0.133	77.5 (65.7‐89.3)	0.136
	Female	58.7 (37.9‐79.5)		58.9 (38.5‐79.3)	
B symptoms	No	92.4 (82.4‐100)	0.005	92.4 (82.4‐100)	0.004
	Yes	58.8 (44.1‐73.5)		60.0 (45.7‐74.3)	
Elevated LDH	No	76.8 (64.6‐89.0)	0.042	77.9 (66.3‐89.5)	0.041
	Yes	57.3 (35.9‐78.7)		58.4 (37.6‐79.2)	
ECOG PS	0‐1	79.8 (74.6‐85.0)	0.000	80.8 (71.0‐90.6)	0.000
	2	0		11.1 (0‐31.7)	
Primary site	Nasal	70.8 (59.4‐82.2)	0.763	71.2 (60.0‐82.4)	0.632
	Non‐nasal	64.3 (23.1‐100)		77.8 (50.6‐100)	
PTI	No	86.0 (71.1‐100)	0.049	87.0 (73.3‐100)	0.038
	Yes	63.6 (49.7‐77.5)		64.5 (51.0‐78.0)	
Ann Arbor stage	I	72.7 (60.4‐85.0)	0.523	74.0 (62.2‐85.8)	0.422
	II	66.7 (44.9‐88.5)		66.7 (44.9‐88.5)	
Regional lymph node	No	68.6 (56.3‐80.9)	0.501	70.1 (58.1‐82.1)	0.563
	Yes	80.0 (59.8‐100)		80.0 (59.8‐100)	
KPI	0‐1	77.9 (65.7‐90.1)	0.045	79.1 (67.5‐90.7)	0.033
	2‐3	57.7 (37.7‐77.7)		57.8 (37.8‐77.8)	
Response to the initial CT	CR	80.6 (63.5‐97.7)	0.223	81.8 (65.7‐97.9)	0.213
	No‐CR	66.5 (53.0‐80.0)		67.7 (54.6‐80.8)	
Response to treatment	CR	81.8 (71.6‐92.0)	0.000	82.9 (73.3‐92.5)	0.000
	No‐CR	23.1 (0.2‐46.0)		23.1 (0.2‐46.0)	

Abbreviations: CT, chemotherapy; KPI, Korean Prognostic Index; LDH, lactate dehydrogenase; PTI, primary tumor invasion.

**Table 3:** Multivariate analysis for PFS and OS

Factor	PFS		OS	
	Hazard radio (95% CI)	*P* value	Hazard radio (95% CI)	*P* value
B symptoms	3.419 (0.736‐15.874)	0.117	4.654 (0.970‐22.321)	0.055
LDH	1.343 (0.457‐3.943)	0.592	1.039 (0.338‐3.187)	0.947
ECOG PS	7.909 (2.085‐30.001)	0.002	10.557 (2.584‐43.132)	0.001
PTI	1.286 (0.322‐5.145)	0.722	1.729 (0.426‐7.007)	0.443
Response to treatment	4.104 (1.212‐13.895)	0.023	3.682 (1.114‐12.164)	0.033

### 3.5 Safety

The major adverse events to GDP regimen were myelosuppression, liver dysfunction, gemcitabine‐related skin rash, blood glucose elevation, and digestive tract toxicities (Table 4). Grade 3 to 4 neutropenia and thrombocytopenia were 18.0% and 15.3%, respectively. Grade 3 nonhematological toxicities observed were skin rash, ALT or blood glucose elevation each in 1 case (1.4%). Mucositis was the most common toxicity during RT. None treatment‐related mortality was observed.

**Table 4:** Toxicity profile

Toxicity	Grade 1/2 (%)	Grade 3/4 (%)
Leucopenia	18 (25.0)	12 (16.7)
Neutropenia	17 (23.6)	13 (18.0)
Anemia	18 (25.0)	0
Thrombocytopenia	9 (12.5)	11 (15.3)
ALT elevation	7 (9.7)	1 (1.4)
Skin rash	3 (4.2)	1 (1.4)
Blood glucose elevation	1 (1.4)	1 (1.4)

## 4 DISCUSSION

The treatment of early stage ENKL has been greatly improved during the past decades with the 5‐year OS increased from 34% to 89%.^3^ Previous studies showed that addition of CT to RT conferred survival advantage for high risk patients.^24^ However, the standard therapy has not yet been established for early‐stage ENKL, including the optimal combination and sequence of RT and CT and standard CT regimen. Concurrent chemotherapy and sequential chemoradiotherapy (SMILE regimen) for localized ENKL are suggested by current guideline from the National Comprehensive Cancer Network (NCCN), but these strategies were seldom applied in our clinical practice because of inconvenience and intolerance. Early or up‐front RT at doses of ≥54 Gy combined with CT was associated with better survival outcomes for localized ENKL,^25^ but so far there is no evidence of a significant survival difference between up‐front RT and early RT for stage II ENKL. Zang et al reported a better outcome with early RT than late RT even combined with L‐asparaginase‐containing CT for early‐stage ENKL.^26^ Therefore, RT is the backbone of curative intent for early‐stage ENKL and should not be delivered too late than up to 4 to 6 cycles of CT.

Sandwich chemoradiation to ENKL was first reported by Agustin Avilés and his colleagues, 32 patients with nasal NK/T‐cell lymphoma and disseminated disease were treated with 3 cycles of CMED followed by IFRT and then 3 cycles of CT.^27^ The 5‐year OS was up to 65% suggested that would be a feasible and effective therapeutic strategy. In clinical settings, many ENKL patients have high fever, skin necrosis and/or poor physical fitness when diagnosed, who may be not suitable for radiotherapy firstly. Meanwhile, initial CT may improve symptoms and performance status instantly and eliminate potential distant lesions. Thus, we believed that sandwich chemoradiation was a promising treatment option for early‐stage ENKL. Recently, there have also been several studies from China applying the sandwich protocol to treat newly diagnosed stage I/II ENKL, with different CT regimens (summarized in Table 5).

**Table 5:** Selected studies on the treatment of newly diagnosed Early‐Stage (IE/IIE) Extranodal Natural Killer/T‐Cell Lymphoma, Nasal Type

Study	Therapy	N	Age (range), y	Initial CT ORR (CR), %	ORR (CR), %	PFS	OS	Comment
Yamaguchi et al^15^, ^28^, ^29^ prospective	Concurrent RT with 3 cycles 2/3DeVIC	27	Median 56 (21‐68)	NA	81 (77)	2‐y, 67% 5‐y, 63%	2‐y, 78% 5‐y, 70%	Grade 3/4 adverse events: leukopenia 100%, neutropenia 93%, anemia 15%, thrombocytopenia 11% and FN 15%; 30% mucositis, 15% dysphagia, and 4% dermatitis due to RT
Jiang et al^15^ Zhang et al^16^ prospective	LVP with sandwiched RT for 4 to 6 cycles	26	Mean 43.5 (18‐74)	92.3 (42.3)	88.5 (80.8)	2‐y, 80.6% 5‐y, 64%	2‐y, 88.5% 5‐y, 64%	7% Grade 3 leukopenia, 23% Grade 3 mucositis, and 11% dermatitis due to RT
Zhang et al^17^ retrospective	CT (CHOP, EPOCH, ATT GELOX et al) with sandwiched RT for 6 cycles	121	Median 40 (19‐68)	87.8 (69.4) (average 2.92 cycles, range 1‐4)	92.6 (90.9)	5‐y, 74.7%	5‐y, 77.3%	Grade 3/4 adverse events: neutropenia 17.4%, FN 7.4%, thrombocytopenia 5.8%, nausea/emesis 6.6%
Wang et al^18^, ^30^ prospective	GELOX† with sandwiched RT for 4 to 6 cycles	27	Mean 47 (21‐74)	92.6 (55.6)	96.3 (74.1)	2‐y, 86% 5‐y, 74%	2‐y, 86% 5‐y, 85%	Grade 3/4 adverse events: Leukopenia 33.3%, thrombocytopenia 29.6%, anemia 7.4%, anorexia 14.8%, decreased fibrinogen 14.8%, vomiting 11.1%, nausea 7.4%, hyperbilirubinaemia 7.4%; Grade 3 radiation‐related mucositis 15%
Wei et al^31^ retrospective	P‐Gemox with sandwiched RT for 4 cycles	35	NA	91.4 (37.1)	94.3 (80.0)	2‐y, 77.1%	2‐y, 82.9%	Grade 3 toxicities were few; 11.4% patients experienced grade 4 toxicities
Xu et al^32^ prospective	MESA with sandwiched RT for 4 cycles	40	Median 49	92.1 (71.1)	92.1 (89.5)	2‐y, 89.1%	2‐y, 92.0%	Grade 3/4 adverse events: nonhematologic toxicities 42.5%, hematologic toxicities 65.0%, and nonhematologic toxicities 22.5% during RT
This study retrospective	GDP with sandwiched RT for 4 to 6 cycles	72	Median 52.5 (21‐79)	91.7 (30.6)	91.7 (81.9)	5‐y, 70.9%	5‐y, 72%	Grade 3/4 adverse events: neutropenia 18.0%, thrombocytopenia 15.3%

Abbreviations: CR, complete remission; CT, chemotherapy; DeVIC, dexamethasone, etoposide, ifosfamide, and carboplatin; GDP, gemcitabine, dexamethasone, and cisplatin; GELOX, gemcitabine, L‐asparaginase, and oxaliplatin; GELOX†, Pegaspargase n=7 and L‐asparaginase n=20; LVP, L‐asparaginase, vincristine, and prednisolone; MESA, methotrexate, etoposide, dexamethasone, and pegaspargase; N, number of patients; NA, not available; ORR, overall response rate; OS, overall survival; OS, overall survival; PFS, progression free survival; P‐Gemox, gemcitabine, oxaliplatin, and pegaspargase; RT, radiotherapy.

It is generally agreed that the conventional anthracycline‐based chemotherapies yield to poor clinical outcomes in the treatment of ENKL related to overexpression of P‐glycoprotein.^7^ Asparaginase‐based or pegaspargase‐based regimens provided favorable outcomes in newly diagnosed and relapse/refractory ENKL, nasal type.^8^, ^9^ Several phase II studies with asparaginase‐based or pegaspargase‐based regimens combined with early RT has been shown to be effective for the treatment of newly diagnosed stage I/II ENKL.^16^, ^17^ However, the toxicity profiles of above regimen limited its wide range of clinical application, especially for early‐stage ENKL. In a retrospective observational study of 41 newly diagnosed stage IV and relapsed/refractory ENKL, GDP regimen resulted in an ORR of 83.0% (CR in 81%).^14^ The 1‐year PFS and OS rates were 54.5% and 72.7%. Grade 3 to 4 adverse events included neutropenia (34.1%), thrombocytopenia (19.5%), and anemia (14.6%).^14^ In a retrospective study, 44 patients with newly diagnosed, stages I/II ENKL treated with intensity‐modulated radiation therapy (IMRT, 50‐56 Gy) followed by GDP chemotherapy, the 3‐year PFS and OS rates were 77% and 85%.^33^ The common grades 3 to 4 adverse events included leukopenia (37%), neutropenia (34%), and mucositis (25%).^33^ These data have showed GDP regimen was also effective for early‐stage patients when integration with appropriate radiotherapy. In fact, GDP regimen could rapidly control the symptoms especially for high fever which was up to 62.5% in our study. The comparable PFS (5‐year: 70.9%) and OS (5‐year: 72.0%) of sandwich chemoradiation with GDP regimen were achieved for early‐stage ENKL, while the toxicities of sandwich chemoradiation with asparaginase‐based or pegaspargase‐based CT were more frequent and serious (summarized in Table 5). It was observed that grade 3/4 adverse events occurred more frequently, including hematologic toxicities and some specific nonhematologic, such as anorexia, hyperbilirubinaemia, decreased fibrinogen and so on. The above data indicated that the tolerability of sandwich chemoradiation with GDP was better than asparaginase‐containing regimens while maintaining similar therapeutic effects.

It is noticeable that the initial CR rate (30.6%) in this study was lower than the study with median 6 cycles of GDP (41.5%), but with comparable ORR (91.7% vs 83%).^14^ The CR rates after 4 to 6 cycles of L‐asparaginase‐containing CT for localized ENKL varied from 60.4% to 81.6%, with ORR rates 84.2% to 100%.^34^, ^35^, ^36^ However, in the retrospective study of sandwich chemoradiation with P‐Gemox regimen for newly diagnosed NK/T‐cell lymphoma, the CR rate for newly diagnosed ENKL was 33.3% at interim and 60.4% on completion; the ORR was 83.3% and 90.6%, respectively.^37^ These prove that the use of more cycles of CT are associated with higher CR rates. The initial CR rates in these sandwich chemoradiation studies varied from 37.1% to 71.1% with comparable ORR. Although, initial CR rates were relatively lower, the CR rates reached as high as 74.1 to 90.9% after radical RT in 6 studies (Table 5). The toxicities of GDP were even less than those in the aforementioned study reported by Wang et al, partly because the patients are initial treated and with localized disease.^14^ This study showed GDP regimen with sandwiched RT had a comparable OS and PFS with other asparaginase‐containing regimens in sandwich pattern. A probable explanation for this phenomenon is that early RT eliminates the negative impact of tumor response between different CT regimens.

ENKL is a heterogeneous disease with different clinical course and treatment outcome. Scholars had tried to find the accurate assessment of poor prognostic factors for discriminating patients at high and low risk of relapse. A Korean group proposed 3 original prognostic scoring system, including the KPI in 2006,^20^ Prognostic Index of Natural Killer Lymphoma (PINK), and PINK‐E (addition of peripheral blood EBV‐DNA status) in 2016.^38^ EBV‐DNA detection was not carried out in our institution while this treatment strategy applied, thus it is a limitation of this study. Four independent factors associated with OS were identified in KPI model: presence of B symptoms, advanced stage, elevated serum LDH level, and regional lymph node involvement. However, B symptoms, elevated serum LDH level, and lymph node involvement were not significant prognostic factors by multivariate analyses in our study. PTI was reported as a novel independent predictive factor for ENKL.^39^, ^40^, ^41^ In our study, presence of PTI was associated with poor OS and PFS in univariate analysis but there was no significant difference in multivariate analyses, maybe due to small sample size. ECOG PS and response to whole treatment were identified as predictors of OS and PFS in this study. Several studies have found that tumor response to initial CT is associated with patient prognosis.^15^, ^17^ In contrast, this study showed the response to initial CT was not predicted outcome, but achievement of CR at completion treatment was associated with better PFS and OS. Similarly, in the study of sandwich chemoradiation with GELOX regimen (gemcitabine, L‐asparaginase, and oxaliplatin), patients who attained a CR at the end of treatment had significantly longer PFS and OS than other patients. In a subgroup analysis of sandwich chemoradiation with LVP regimen (L‐asparaginase, vincristine, and prednisolone), the 5‐year OS rates were higher for patients who achieved a CR (76% compared to 0% for those without a CR).^16^ Achievement of CR at completion treatment is an independent prognostic factor for early‐stage ENKL, but the survival benefit should be demonstrated in a large randomized study.

## 5 CONCLUSIONS

In conclusion, this study provides evidence to confirm sandwich chemoradiation with GDP regimen as highly effective treatment with markedly less toxicities in ENKL, especially for those having fever and/or extensive lesions. It may be considered as a good treatment option for patients with newly diagnosed stage I/II ENKL. Larger, prospective randomized clinical trial should be performed to further confirm those findings.

## CONFLICT OF INTEREST

The authors declare no conflict of interest.

## AUTHOR CONTRIBUTIONS

Study concepts, Hao Ding and Xichun Hu; Study design, Hao Ding and Shu Tian; Data acquisition, Shu Tian, Hao Ding, Tian Wang, Shengzi Wang, and Rong Tao; Quality control of data and algorithms, Shu Tian and Hao Ding; Data analysis and interpretation, Shu Tian, Ruichen Li; Statistical analysis, Shu Tian and Ruichen Li; Manuscript preparation, Shu Tian; manuscript editing and review, Hao Ding and Xichun Hu; Funding acquisition, Shu Tian.
